# A national survey of autoimmune glomerular disease services in the UK

**DOI:** 10.1186/s12882-026-05257-6

**Published:** 2026-08-05

**Authors:** Lucy Francis, Jonathan Barratt, Jane Hollis, Lisa Willcocks, Rachel B. Jones

**Affiliations:** 1 Department of Medicine, University of Cambridge, Cambridge, UK; 2 Department of Nephrology, Addenbrooke’s Hospital, Addenbrooke’s Vasculitis and Lupus Department, Cambridge University Hospitals NHS Trust, Hills Road, Cambridge, CB2 0QQ UK; 3 John Walls Renal Unit, University Hospitals of Leicester NHS Trust, Leicester, UK

**Keywords:** Glomerulonephritis, Rare disease, Regional multidisciplinary teams, Workforce capacity, Nephrology services, Research infrastructure, Novel therapeutics, National survey, Autoimmune glomerular disease

## Abstract

**Background:**

Glomerulonephritis (GN) accounts for 20%–25% of chronic kidney disease and is a major cause of end-stage kidney disease. The 2021 UK Rare Disease Framework highlighted inequities in rare disease care, with commitments to integrated care and equitable access. This national survey explored unmet needs in GN services, particularly service delivery, access to novel therapies, and research opportunities.

**Methods:**

An online survey was designed by the UK Kidney Research Consortium Glomerulonephritis Clinical Study Group and distributed to UK adult and paediatric nephrologists. The 25-item survey assessed service infrastructure and staffing, equity of access to novel therapies, and research infrastructure and barriers. Responses were analysed using descriptive statistics and qualitative content analysis.

**Results:**

Eighty-five responses were received from all eight NHS England renal networks and from Northern Ireland, Scotland, and Wales, including 73 adult respondents from 57 renal centres and 12 paediatric nephrologists from 10 paediatric centres. After removing duplicate responses per centre, 67% (38/57) of adult respondents from 57 centres reported working in a tertiary renal centre. Overall, 67% (38/57) reported a dedicated primary glomerulonephritis, vasculitis or lupus clinic, 49% (28/57) cohorted patients by disease subtype, and 32% (18/57) ran more than one clinic per week. Most reported access to day-case infusion services 93% (53/57), plasma exchange 77% (44/57), and in-house multidisciplinary teams 74% (42/57). There was a reported absence of a specialist clinic in 33% (19/57) of centres, in-house glomerular disease specialist consultant support 30% (17/57), specialist nurses 61% (35/57), and renal research nurses 18% (10/57). Regional multidisciplinary meetings and dedicated patient messaging systems were unavailable for 58% (33/57) and 60% (34/57) respectively. In England, access to rituximab, avacopan, belimumab and voclosporin was variable. Research activity was reported by 72% (41/57) for commercial and 56% (32/57) for non-commercial studies. Most respondents 96% (55/57) expressed a desire to increase research involvement, and 81% (46/57) wished to lead their own studies. Reported barriers in embedding research into clinical practice included lack of clinician time, insufficient job-plan allocation, limited research nurse availability and funding. Paediatric respondents also reported similar constraints.

**Conclusions:**

This survey highlights disparity in access to specialist clinics, specialist nurse support and regional MDTs in glomerular disease across the UK. Nephrologists remain highly motivated to conduct research, though capacity and funding constraints persist.

**Clinical trial number:**

Not applicable.

**Supplementary Information:**

The online version contains supplementary material available at https://doi.org/10.1186/s12882-026-05257-6.

## Background

In the UK, the prevalence of patients with kidney disease is expected to reach 7.6 million by 2033. Current estimates suggest an annual economic cost of approximately £7 billion, of which £6.4 billion relates directly to National Health Service care. Without meaningful intervention, total costs could rise to almost £14 billion within the next decade [1]. Glomerulonephritis (GN) accounts for approximately 20–25% of chronic kidney disease cases in most renal registries and remains a leading cause of end-stage kidney disease [2].

Major subgroups of GN include systemic diseases such as small-vessel vasculitis and lupus nephritis (LN), and primary glomerular conditions that include IgA nephropathy (IgAN), membranous nephropathy, minimal change disease, focal segmental glomerulosclerosis, and C3 glomerulopathy. Accurate recognition of disease mechanisms, stage of progression, and specific subtype is essential to guide optimal treatment.

Recent progress in understanding of disease pathogenesis, alongside regulatory changes and a growing clinical trial pipeline, has led to the development of more targeted immunosuppressive therapies aimed at specific immune pathways [3]. As insight into pathogenesis grows, treatment strategies are shifting focus from managing chronic kidney disease and delaying common pathways of progression to end-stage renal failure, toward earlier intervention with targeted immunomodulatory therapies aimed at preventing irreversible glomerular damage in individual glomerular diseases [2]. These developments are reshaping clinical practice, and are likely to place additional demands on specialist GN services.

In the UK, recent therapy approvals include belimumab for systemic lupus erythematosus, voclosporin for LN, and targeted-release budesonide and sparsentan for IgAN, with obinutuzumab for LN currently under regulatory review [4–8]. Multiple agents are also currently in phase 3 clinical trials across the spectrum of glomerulonephritides [9–11]. The pace of innovation is such that CAR-T cell therapies and bi/tri-specific antibodies are now entering trials in SLE, LN, AAV and other autoimmune diseases [12, 13]. These advances signal a transformative shift in the therapeutic landscape for renal autoimmune diseases, an evolution expected to continue over the next decade.

The rapid emergence of novel therapies has begun to outpace existing treatment guidelines, necessitating successive updates to clinical practice recommendations (e.g. Kidney Disease: Improving Global Outcomes (KDIGO) 2024 Lupus Nephritis; KDIGO 2024 ANCA-Associated Vasculitis; KDIGO 2025 IgA Nephropathy and IgA Vasculitis) [14–16]. With increasing immune-mediated complexity and the pace of therapeutic innovation, these diseases demand increasingly specialist expertise to keep abreast of evolving treatment paradigms.

Alongside the rapid progress with targeted treatment development, renal service structures and networks will need to adapt at pace; particularly with respect to clinician education, care pathways, and equitable access to emerging novel therapeutics, and cross-speciality working for the multisystem diseases. In England, adult renal services are provided by 51 trusts operating in a hub and spoke model, including 19 acute transplanting centres. In 2021, NHS England set up eight renal clinical networks to coordinate patient pathways between centres over a wide area to ensure access to specialist resources and expertise, supported by the Renal Services Transformation Programme. Renal networks aim to improve the quality and equity of care for renal patients in their catchment population encompassing the whole adult renal patient pathway. This has included a number of workstreams, but with no explicit consideration of glomerulonephritis management [17].

In paediatrics, similar challenges are evident. There are approximately 1,000 children with severe CKD (< 30 mL/min/1.73 m^2^) in the UK [18]. Specialist care is delivered through 13 tertiary paediatric nephrology centres [19]. Improving access to local care through regional paediatric nephrology networks supported by general paediatricians with a special interest in nephrology (SPIN) was identified as a priority in a 2011 report from the Royal College of Paediatrics and Child Health, British Association for Paediatric Nephrology & NHS Kidney Care [20]. Despite this, infrastructure to support widespread networked care remains limited. Therapeutic complexity adds further challenges, as many treatments for paediatric renal autoimmune disease require off-label use, renal-specific dose adjustments, and experience that extends beyond routine paediatric practice.

In contrast to adult and paediatric NHS renal services, rheumatology have had a more established framework for rare autoimmune disease for over a decade, supported through NHS England specialised commissioning [21]. Nephrology currently lacks an equivalent structure for rare renal autoimmune disease, despite a rapidly evolving therapeutic landscape and increasing complexity of care. The 2024 UK Parliamentary review on rare autoimmune rheumatic diseases explicitly recommended a network-based approach to ensure equitable access to multidisciplinary expertise; however, a parallel system for renal autoimmune disease within existing renal networks remains undefined [22].

At a broader level, the 2021 UK Rare Disease Framework emphasised earlier diagnosis, improved coordination of care, and equitable access to specialist centres for patients with rare disease [23]. Building on these national priorities, the 2025 British Society for Rheumatology management recommendations for AAV introduced an AAV service specification that emphasises timely access to expert review and treatment, integrated multidisciplinary care through cohorted and nurse-led clinics, and equitable access to specialist MDTs for coordinated management and approval of novel therapies [24]. Within this specification, a specialist centre is defined as one providing MDT input for at least 50 patients with AAV, supporting the adoption of new therapeutics and contributing actively to research.

Despite evolving frameworks, there remains limited national data on existing service provision for GN services across the UK. In particular, the availability of specialist renal autoimmune nurses, equitable access to regional MDTs, and the prescribing capacity of renal pharmacists experienced in novel immunotherapies are all poorly characterised. There has also been a large but underserviced kidney research agenda in the UK. The Kidney Research Consortium (KRC) was established in recognition of this in 2007. A research strategy was published in 2016 [25]. Under the umbrella of the KRC, 16 clinical study groups (CSG) exist to drive the research agenda within subspecialist areas. The glomerulonephritis (CSG) group seeks to drive clinical research across GN in the UK and whilst an informal group, comprises a mailing list of 130 consultants with an interest in GN. In recognition of the pace of commercial clinical research and absence of commissioned services for GN within renal medicine, the UK KRC GN Clinical Study Group, set out to take a snapshot survey of services across the UK to understand variation in services and identify unmet needs.

## Methods

This online survey was developed by members of the UK KRC GN CSG and developed and administered using Jisc Online Surveys, a secure platform used for academic research in the UK. The survey included both closed-ended questions (multiple choice and binary questions) and open-ended free-text fields. The survey contained 25 questions around three key areas: (1) infrastructure and staffing for renal autoimmune disease service delivery, including specialist clinics, workforce and MDT access; (2) equity of access to novel therapies for renal autoimmune disease; and (3) perceptions of research infrastructure, activity and barriers to clinician-led research. Closed-ended questions used standardised response formats, including single- and multi-select drop-down lists (e.g., regional network, specialist clinic type, Blueteq access) and binary yes/no items specific to primary GN, vasculitis and lupus services (e.g., plasma exchange, MDT access, infusion service, clinical trial participation). Binary questions were supplemented with optional free-text fields to capture further detail.

The survey link was made available from July 2023 to March 2024 to adult and paediatric consultant nephrologists involved in the care of patients with renal autoimmune disease. It was distributed nationally via the UK KRC GN CSG mailing list. Consultant nephrologists at NHS centres not represented in the mailing list were contacted directly to maximise national participation. The survey was also shared with SPIN paediatricians and paediatric nephrologists. Eligible participants included those based in tertiary and non-tertiary renal centres. A formal definition of a ‘tertiary centre’ was not provided. However, in the UK it is widely accepted that a tertiary centre comprises specialist expertise and infrastructure to manage acute kidney injury in critically unwell patients as well as offer specialist services such as renal transplantation. Typically, a tertiary renal centre is a large teaching hospital that acts as research and teaching hubs. Participation was voluntary, not incentivised, and responses could be anonymised. Where multiple responses were received from the same centre, the most complete submission or the response provided by the GN lead was selected.

Free-text responses were analysed using inductive content analysis by a single author, who identified recurring codes and grouped these into overarching themes. Ambiguous responses were discussed with a second author to reach consensus.

## Results

**Fig. 1:**
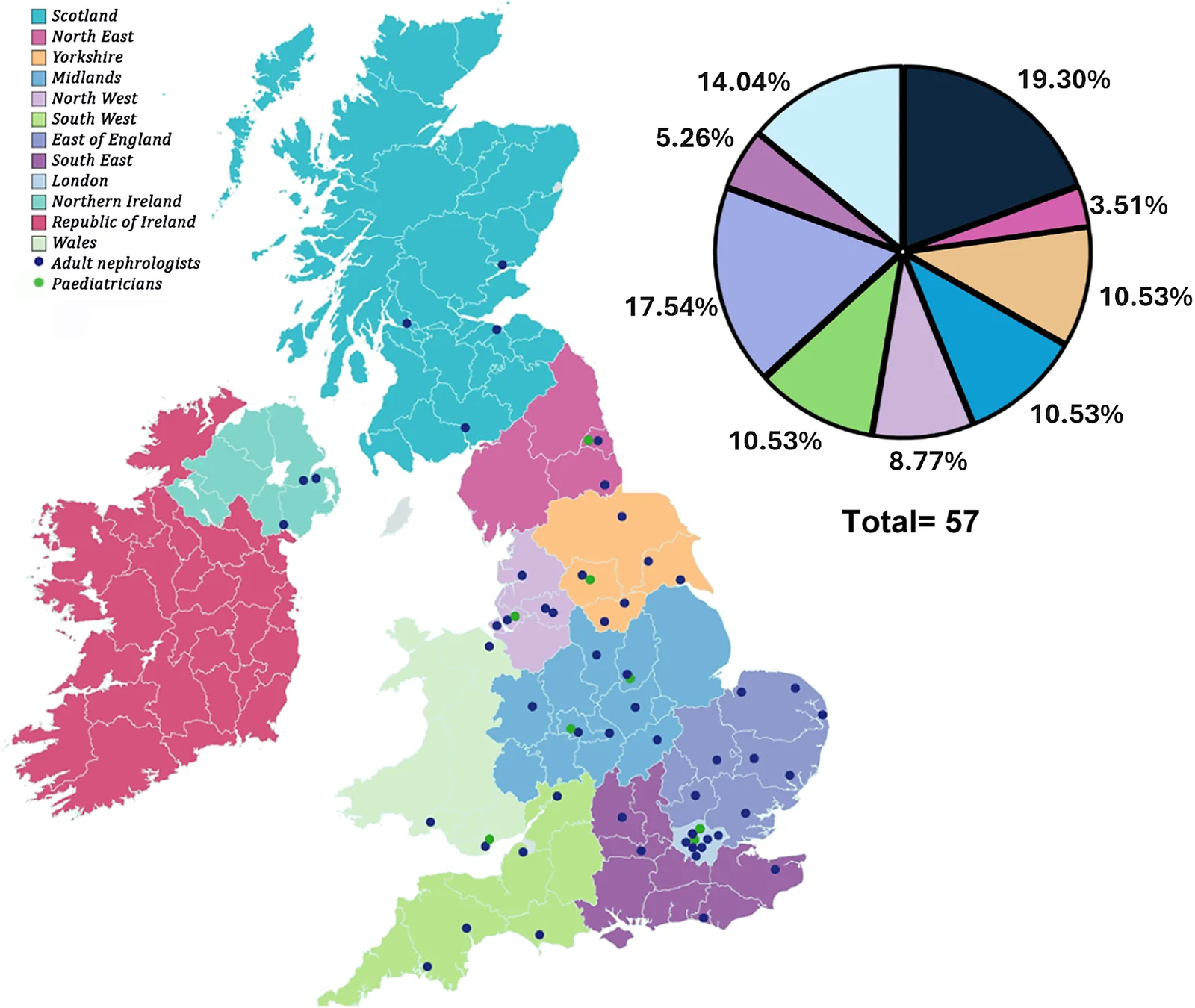
Survey responses on autoimmune glomerular disease services across the UK. On the left is a map of adult nephrology and paediatric survey responses. Each dot represents a responding nephrologist (adult, blue; paediatric, green) by centre. Colours denote the eight NHS renal networks, Scotland, Wales, Northern Ireland, and the Republic of Ireland. Created using www.mapchart.net. On the right is a pie chart of response rate by regional network. After removing duplicate responses from the same centre, 73 individual responses yielded 57 unique responding renal centres. Devolved nations are combined and shown in the darkest blue

**Fig. 2:**
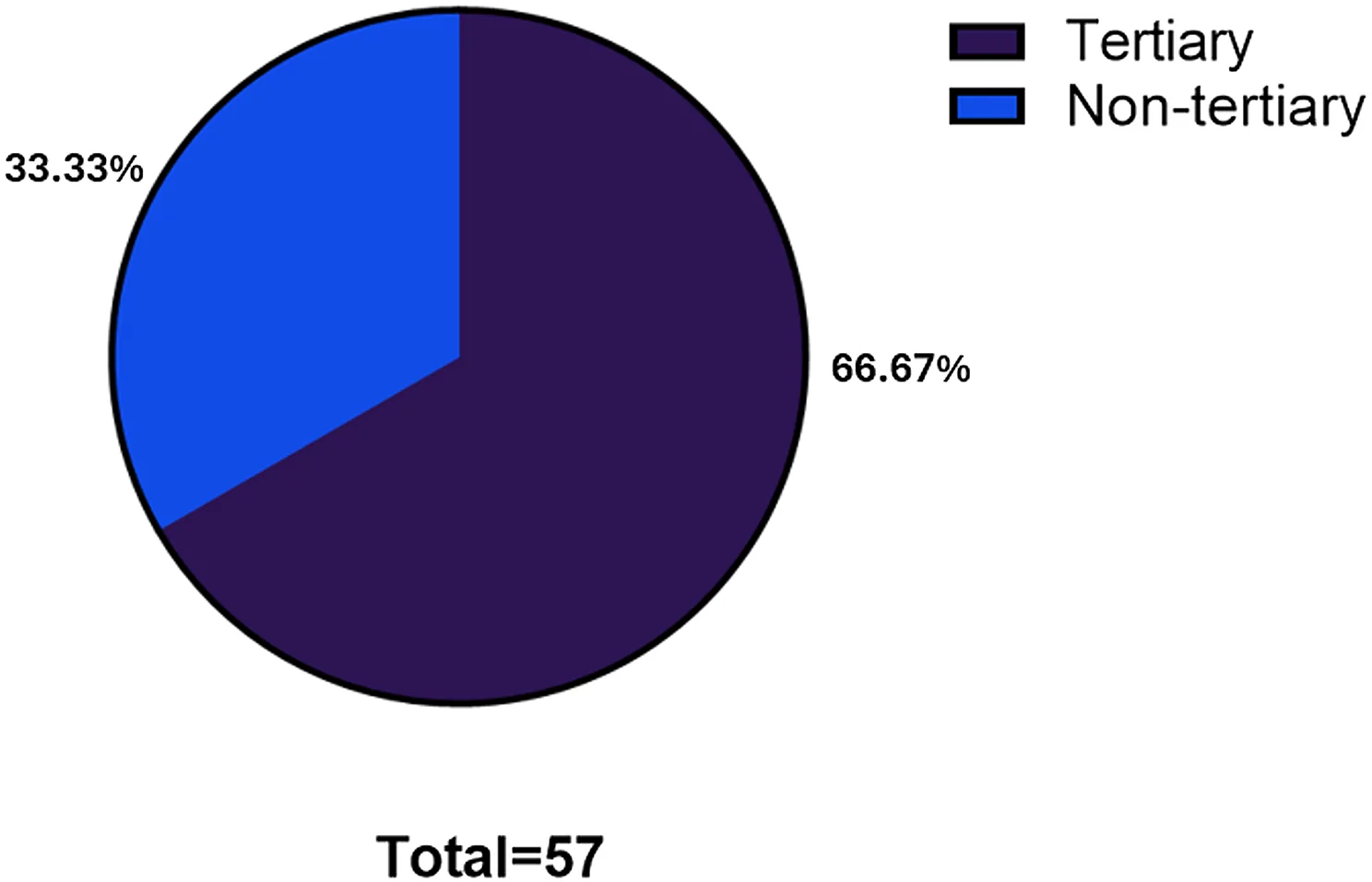
Classification of adult renal centres by tertiary status. Pie chart of adult nephrologists self-reporting whether their centre was tertiary or non-tertiary. Duplicate responses from the same centre were removed, yielding a total of 57 unique renal centres from 73 individual responses

Eighty-five responses were received from 73 adult nephrologists from 57 UK renal centres and 12 paediatric nephrologists from 10 paediatric centres. Responses spanned the eight NHS England renal networks, Northern Ireland, Scotland, and Wales (Fig. 1). Of the 73 nephrologists managing adults, 30% were female (*n* = 22), 68% male (*n* = 50) and 1.3% unknown (*n* = 1). After removing duplicate responses per centre, amongst adult respondents, 67% (38/57) reported working in a tertiary renal centre (Fig. 2). With one response per centre, 25% were female respondents (14/57) and 75% were male (43/57). Nine respondents from the 57 centres self-identified as clinical director or clinical lead.

This national survey highlights substantial variation in the delivery of renal autoimmune disease services across the United Kingdom (Table 1). Specialist nursing support and a dedicated advice phone line or patient messaging system were most commonly reported as absent (61% and 60%, respectively), with similar findings across tertiary and non-tertiary centres. Access to day-case infusion services, plasma exchange and in house MDTs were high nationally. 28/57 centre responses specified that other specialities participated in their MDTs, 82% (23/28) included Rheumatology and 43% (12/28) included Respiratory. Three responses noted additional specialty input from immunology, pathology, and ear, nose and throat doctors. Where respondents reported no access to plasma exchange, 85% identified as being from non-tertiary units, as did 67% of those with no in-house MDTs and 65% of those lacking glomerular disease specialist consultant support. These disparities may be contributing to inequities in patient care.

**Table 1:** Summary of responses from adult respondents from 57 renal centres regarding service features (one response per centre included)

Domain	Service feature	Percentage of responses reporting access to service feature, % (*n*/57 centres)	Respondents identifying their centre as non-tertiary, as a % of the access/no access subgroups (*n*)
Clinic organisation	Dedicated primary GN/vasculitis/lupus clinic	67% (38/57)	• Access: 23% (9/38) • No Access: 58% (11/19)
	Cohorting by disease subtype	49% (28/57)	29% (8/28)
	More than one clinic per week	32% (18/57)	6% (1/18)
Service and in-house MDT access	Day-case infusion services	93% (53/57)	• Access: 30% (16/53) • No access: 75% (3/4)
	Plasma exchange services	77% (44/57)	• Access: 18% (8/44) No access: 85% (11/13)
	In-house multidisciplinary team	74% (42/57)	• Access: 21% (9/42) • No access: 67% (10/15)
Reportedly unavailable	No specialist clinic	33% (19/57)	53% (10/19)
	No specialist nurse support	61% (35/57)	43% (15/35)
	No renal research nurse support	18% (10/57)	50% (5/10)
	No glomerular disease specialist consultant support	30% (17/57)	65% (11/17)
	No regional MDT	58% (33/57)	27% (9/33)
	No dedicated patient messaging system	60% (34/57)	44% (15/34)

Of the 73 adult nephrologist responses, 60 provided unique free-text comments on how their service could be improved. For this qualitative analysis, all free-text responses were used and multiple responses from a single centre were not excluded.

Eleven recurring themes were identified from the free-text comments (Table 2). The changing treatment landscape was recognised by respondents, with one commenting that there were “clearly lots of new medication for IgA/vasculitis/LN.” The most frequently highlighted needs were specialist nursing workforce (32/60) and MDT/regional level collaboration (19/60, Table 2). Free-text responses also identified perceived deficits in organisational and funding support, including protected programme activities (11/60), and in infrastructure and treatment-delivery capacity, such as clinic and infusion space (8/60).

**Table 2:** Thematic analysis of free-text responses from 60 adult nephrologists on service improvement needs

Theme	Number of responses from 60 unique free-text responses	Number of networks or devolved regions mentioning theme	Top three networks or devolved regions mentioning theme by frequency, *n*	What would help improve your service? (2 examples of free text responses)
Specialist nursing workforce	32/60	10	• London, 8 • North West, 7 • Midlands, 4	• “We are losing our nurse which was charity supported – and this will not be replaced by the trust.” • “A specialist nurse with a telephone line to provide advice to patients will really help.”
MDT and regional level collaboration	19/60	9	• East of England, 3 • North West, 3 • Yorkshire, 3	• “Recognition at a trust level for regional MDTs” • “A regional network group for vasculitis, GN and lupus is urgently needed”
Dedicated specialist clinics	11/60	7	• East of England, 3 • South West, 2 • Scotland, 2	• “Dedicated clinics would likely help focus and improve patient pathways” • “I started running the specialist clinic in my spare time due to need. It has been a challenge to have my trust recognise the importance of this clinic.”
Organisational and funding support (including programmed activity time)	11/60	8	• South West, 2 • Yorkshire, 2 • London, 2	• “Money, people, time, space, recognition from managers” • “Job planned time to develop service”
Infrastructure and treatment-delivery capacity	8/60	5	• Midlands, 4 • North West, 1 • North East, 1	• “More capacity in our infusion suite” • “More rooms for outpatient service”
Consultant workforce	7/60	5	• South West, 2 • North West, 2 • East of England, 1	• “A third consultant to help with our patient load” • “Specialist consultants are being pulled to provide general cover and not allowed to prioritise this group of unwell patients”
Specialist pharmacist workforce	7/60	5	• North West, 2 • East of England, 2 • Midlands, 1	• “Additional specialist pharmacy support, the evolving therapeutic options are great for patients but the service evolution has placed an increasing burden on our specialist pharmacy staff and our commissioning models have not kept pace with the clinical developments in vasculitis management” • “We are trying to get a region wide pharmacist role to facilitate Blueteq applications” etc.
Administrative support	4/60	4	• North West, 1 • North East, 1 • Midlands, 1	• “Admin support for MDT” • “More dedicated admin support”
Quality assurance and standards	4/60	2	• Scotland, 3 • North West, 1	• “More resource for proper audit, participation in national audit” • “a regional network group, to allow regional quality improvement work ”
Training and education	3/60	3	• Midlands, 1 • Scotland, 1 • South West, 1	• “Holistic resources for the patients and opportunity to teach for succession planning” • “We are looking for avenues to train/educate as well”
Innovative and community based care	2/60	2	• East of England, 1 • North West, 1	• “Access to remote bloods in primary care via a diagnostic hub to enable bloods to happen prior to clinic appointments.” • “More patients on home rituximab”

Blueteq NHS is a web-based software system used by the NHS in England since 2008 and in Wales since 2021 to manage the authorization and prescribing of novel therapeutics. It ensures that these medicines are prescribed in line with national guidelines from organizations like the National Institute for Health and Care Excellence (NICE). For many novel therapies, national guidelines and Blueteq approval mandate MDT discussion before prescribing can occur. Therefore both access to a specialist glomerular disease MDT and access to the electronic Blueteq system may be barriers to Blueteq approval within individual hospitals in England and Wales.

**Fig. 3:**
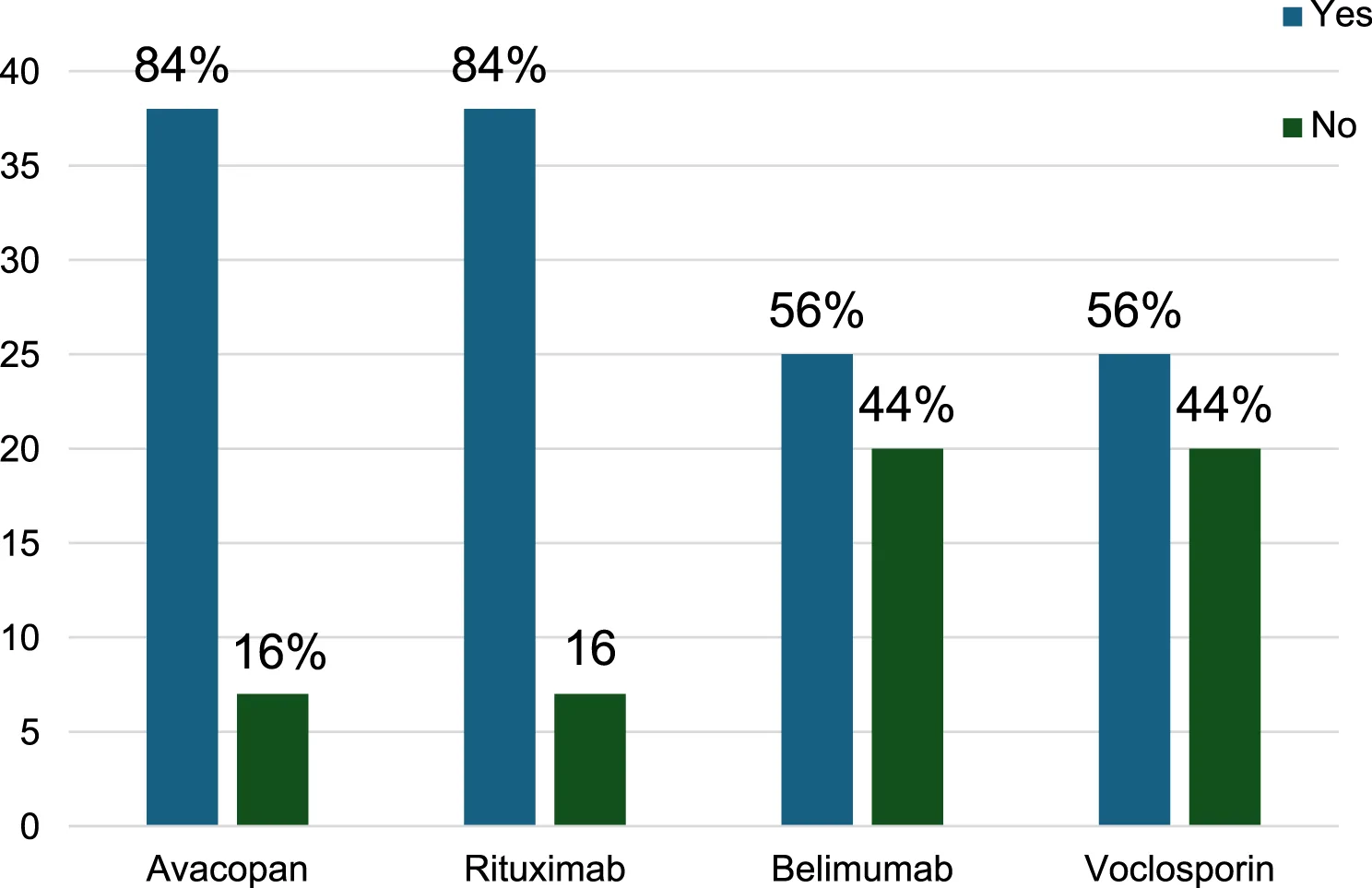
Survey responses from the eight renal networks in England on Blueteq access for the following novel therapeutics: avacopan, rituximab, belimumab, and voclosporin, July 2023 to March 2024. Clustered bar chart with responses reporting access to a novel therapeutic agent in blue, and no access to a novel therapeutic agent in green (n = 45). NICE approved use of avacopan for AAV in 2022, rituximab for AAV in 2014, belimumab for SLE in 2021 and voclosporin for LN in 2023

Including only one response per centre, 45 adult survey respondents from 46 centres from the eight renal networks in England provided information on the Blueteq approval process. Wales was not included given the much more recent introduction of Blueteq. While the majority of respondents working in England reported having approval pathways in place for rituximab and avacopan (both approved for AAV), access to belimumab (approved for SLE, without explicit approval for LN) and voclosporin (NICE approved for LN within 12 months of this survey), was more variable, highlighting inconsistencies in availability across England (Fig. 3). Access appeared to be lowest in the East of England and North East networks however these estimates are based on a very small numbers of centres (*n* = 10 and *n* = 2, respectively) and should be interpreted cautiously. Among the 45 English centres providing Blueteq information, those without access to belimumab were more likely to lack an in-house MDT (40%, 8/20) and specialist nurse support (80%, 16/20); than centres with access (8%, 2/25 and 40%, 10/25, respectively). A similar pattern was seen for voclosporin. Centres without access to voclosporin were more likely to lack an in-house MDT (45%, 9/20 compared to 4%, 1/25 with access), although specialist nursing absence was common across centres regardless of voclosporin access (60%, 12/20 compared to 56%, 14/25). Since this survey was conducted, the European Medicines Agency has recommended revoking avacopan’s European Union marketing authorisation, though its UK licensing status remains unchanged at the time of writing.

Twenty respondents from 57 centres reported relying on alternative pathways for accessing novel therapeutics across the UK. 11/20 respondents describing other arrangements were working in Scotland, Wales, and Northern Ireland. In Wales, NHS Blueteq access was implemented widely only in 2021 and in Scotland and Northern Ireland, non-NHS devolved healthcare systems exist. Access in these regions was also shaped by national or regional bodies such as the Scottish Medicines Consortium and the Welsh Health Specialised Services Committee. At the local level, drug and therapeutics committees, medical director approval, and occasional chairman’s actions were cited as routes for drug authorisation. Some centres obtained therapies through neighbouring specialist units or regional MDTs, while a small number reported funding selected treatments directly from departmental budgets. Overall, these responses underscore the complexity and variability of approval pathways for novel therapies outside England’s Blueteq system (Table 3).

Despite increasing service pressures, clinicians demonstrated a strong commitment to research in renal autoimmune diseases. A substantial majority of adult nephrologists from 57 centres − 96% (55/57) expressed a desire to expand their involvement in glomerulonephritis (GN) research, with 81% (46/57) specifically interested in establishing their own investigator-led studies.

Of the 73 adult nephrologist responses, 65 provided unique free-text comments on the challenges of conducting research. All free-text responses were analysed, and multiple responses from the same centre were not excluded. 51% of responses from all free-text comments (33/65) cited consultant time and competing clinical workload as limiting factors. Similarly, 31% (20/65) mentioned limitations on research nurse support and time. Comments included “adequate nursing support is challenging” and “we need more support with recruiting specialists nurses at DGH level”. 26% (17/65) cited limited funding specifically. 11% of respondents (7/65) described the administrative burden and bureaucracy of trials. Opportunity constraints from not being invited to participate in trials or having too few patients were cited by 9% (6/65).

Notably, 18% from 57 centres (10/57) had no access to research nurse support for renal autoimmune disease within their institution. Nevertheless, research activity was reported by 72% (41/57) for commercial and 56% (32/57) for non-commercial studies.

The survey also invited clinicians to provide free-text reflections on the positive experiences of engaging in renal autoimmune disease research. Positive comments highlighted strong existing research teams and a high level of enthusiasm among both patients and staff to participate in NIHR and academic studies. Some respondents also noted successful involvement in major national and international trials.

Responses from paediatricians were received from the Great North Children’s Hospital (Newcastle), Evelina London Children’s Hospital, Great Ormond Street Hospital for Children, Leeds Children’s Hospital, Alder Hey Children’s Hospital (Liverpool), Nottingham Children’s Renal and Urology Unit, Birmingham Children’s Hospital, and the University Hospital of Wales. 58% were male respondents (*n* = 7) and 42% female (*n* = 5). There were 12 responses. Following the exclusion of duplicate entries from the same centres, 10 unique responses were analysed.

Paediatric nephrologists reported similar challenges to adult nephrologists with service delivery and integrating research into clinical practice (Table 4).

**Table 3:** Summary of responses from paediatric nephrologists regarding service features (one response per centre included)

Domain	Service feature	Percentage % (*n*/10 centres)
Clinic organisation	Dedicated primary GN/vasculitis/lupus clinic	50% (5/10)
	Cohorting by disease subtype	40% (4/10)
	More than one clinic per week	20% (2/10)
Service and in-house MDT access	Day-case infusion services	80% (8/10)
	Plasma exchange services	70% (7/10)
	In-house multidisciplinary team	90% (9/10)
Reportedly unavailable	No specialist clinic	50% (5/10)
	No specialist nurse support	30% (3/10)
	No renal research nurse support	30% (3/10)
	No glomerular disease specialist consultant support	30% (3/10)
	No regional MDT	80% (8/10)
	No dedicated patient messaging system	50% (5/10)

**Table 4:** Selected free-text comments from paediatric nephrologists on service improvement and research challenges. Given the small number of paediatric respondents (*n* = 10), free-text responses are presented as illustrative groupings of related comments rather than a formal thematic analysis

Free-text response (some examples)	
Specialist nursing workforce	“More specialist nurse, consultant, pharmacy and admin time. More clinic rooms” “More medical and nursing staff”
Dedicated specialist clinics	“More dedicated clinics” “Have a specific clinic for these conditions”
National coordination	“National network for paediatric vasculitis” “A national paediatric inflammatory commissioning service to provide indications/permissions/oversight and equality of access, our complex cases meeting could evolve into this. We have nationally leading paediatric pharmacology service that no other centres have”
Organisational and funding support (including programmed activity time)	“More time and funding” “
Rare disease status limiting trial capacity	“Capacity in paediatric academia. Rare diseases so open and risk not recruiting”
	“Paediatric patient group-limited trials “
Research funding, workforce and delivery capacity	“Time in job plans. Rare paediatric diseases, perceived low value for money”
	“Funding for research PAs”
	“Not much support, no set time for research, busy with clinical duties, research time not considered for additional payment even if it generates money”
Other miscellaneous comments	“More evidence to support therapy”
	“Earlier access to B cell depletion”
	“Transition care”

Key priorities for future service improvement amongst paediatricians included national coordination through a vasculitis network, streamlined commissioning pathways for inflammatory kidney diseases, and earlier, more consistent access to B cell–targeted therapies.

## Discussion

This survey arose out of recognition of the rapid pace of drug development for glomerular diseases and the urgent unmet need for service development to deliver increasingly specialist care. We provide a snapshot of renal autoimmune disease service provision in the UK from June 2023–2024, and highlight significant variation in infrastructure, workforce, and access to therapies for autoimmune rare renal diseases.

While many respondents reported access to infusion centres and plasma exchange, availability of specialist nurses, specialist pharmacist input, and regional MDT meetings for novel therapy approval was less consistent, particularly among non-tertiary centres. Although not a formal comparative study, non-tertiary centres accounted for a disproportionate share of those identifying as lacking plasma exchange (85%), in-house MDT input (67%), and glomerular disease specialist consultant support (65%), despite representing only 33% (19/57) of respondents from 57 centres. Reduced local access to plasma exchange could plausibly affect timeliness of treatment for rapidly progressive disease, and more limited MDT or consultant input may affect the consistency with which guideline-recommended, MDT-dependent treatment decisions are reached. These observations point to areas where standardising GN care across tertiary and non-tertiary settings may warrant attention.

The 2021 Getting It Right First Time (GIRFT) nephrology report identified wide variation in the delivery of renal services both across England and within regions. It recommended improving access to coordinated monitoring, research opportunities, and specialist therapies through better linkage with appropriate expertise and a stronger networked approach [26]. However, the focus of the 2021 GIRFT report was limited to the core commissioned elements of renal care; transplantation, dialysis, chronic kidney disease (CKD), and acute kidney injury.

The Rare Autoimmune Rheumatic Disease Alliance (RAIRDA) has played a central advocacy role in this space, calling for greater equity of access, transparency of data, and improved service integration across the UK’s rare autoimmune conditions [27]. The 2024 Scottish Vasculitis Outcomes in Relation to Care Experiences (VOICES) project also highlighted substantial challenges for patients, including delayed diagnosis and inconsistent access to vasculitis expertise. Importantly, it demonstrated that access to multidisciplinary team (MDT) review among 1,420 patients with AAV was associated with fewer emergency admissions and serious infections compared with those without MDT input [28].

The UK nephrology workforce, however, has faced significant challenges in recent years, exacerbated by the COVID-19 pandemic. A 2021 national survey conducted by the UK Renal Specialist Registrar Club and the UK Kidney Association found that 57% of respondents met criteria for burnout [29]. In parallel, the research capacity of the workforce appears to be contracting. According to the Office for Strategic Coordination of Health Research, between 2012 and 2022 there was a 6% decline in medically trained research staff, including a 24% reduction in the academic posts of senior lecturer [30]. These trends may have important implications for the sustainability of research in renal autoimmune disease.

We acknowledge a likely bias towards survey completion by nephrologists motivated by a specialist interest in glomerular disease. However, such nephrologists lead GN services and are best informed on unmet need in this sub-specialist area. Consistent with this bias, our survey identified strong clinician interest in GN research; the majority expressed a desire to expand their involvement, but the findings also highlighted barriers to participation. Lack of protected time within consultant job plans, limited research nurse support, and insufficient access to funding were major barriers. Similar challenges were reported in a 2022 survey of allied healthcare professionals in renal medicine, where less than half were actively involved in research despite high levels of awareness and enthusiasm. Reported barriers again included workforce shortages, time constraints, and lack of support [31].

The results of this survey also point to several practical areas for service improvement, particularly aimed at supporting earlier intervention before permanent glomerular injury occurs. Strengthening the renal workforce, including expansion of consultant posts and increasing training numbers for nephrology registrars, where feasible, could enable greater flexibility in job planning, including the allocation of dedicated programmed time for glomerular disease care, education, and research.

Greater investment in renal nursing, particularly in specialist autoimmune disease nurses, is essential to support a more preventative approach to managing patients earlier in their disease course. Early specialist input offers the potential to reduce long-term kidney damage by intervening before chronic, irreversible changes occur. The 2024 Scottish Vasculitis Outcomes in Relation to Care Experiences (VOICES) project found that access to cohorted specialist clinics and nurse-led clinics were associated with reductions in emergency hospital admissions among patients with systemic vasculitis [28]. These findings align with an emerging international consensus on structured, specialist-led models of care: a draft Consensus Statement from the International Society of Glomerular Disease has proposed certification standards for Glomerular Disease Centres of Excellence, outlining expectations for clinical expertise, education, research, multidisciplinary practice, and patient advocacy [32].

In paediatrics, children with rare renal conditions remain significantly underrepresented in clinical trials, due to a combination of ethical and regulatory barriers, limited funding, and insufficient research infrastructure—including a shortage of staff trained to deliver paediatric clinical studies [33]. Delivering treatment at home can also be complex, with the need to navigate emotional, social, and educational challenges unique to children [34]. In our survey, 80% reported no access to a regional MDT and 50% reported no specialist clinic. The Royal College of Paediatrics and Child Health has highlighted growing concerns around workforce capacity, recommending a 20% increase in consultant paediatric nephrologists to meet rising clinical demand, reduce regional disparities, and alleviate pressures on tertiary services [35].

Encouragingly, several recent initiatives aim to address these issues in both adult and paediatric nephrology. A dedicated vasculitis specialist nurse/MDT session was introduced at the United Kingdom and Ireland Vasculitis Rare Disease Group (UKIVAS) training course in November 2024, fostering shared learning across centres [36]. In addition, the launch of a three-day training course from the Vasculitis Nurses Academy at Cambridge University Hospitals in June 2025 represents a promising step toward equipping nurses with the advanced skills needed to manage complex autoimmune kidney diseases. The course immediately attracted 27 expressions of interest for specialist nurse training from 19 NHS Trusts across the UK upon initial advertisement, underscoring the clear national demand [37].

Expanding regional autoimmune MDTs within existing renal networks may also help formalise clinical pathways, facilitate consistent recommendations for novel therapeutics, and reduce regional variation in access, improving equity and outcomes for patients nationally. Embedding the role of specialist pharmacists in regional renal autoimmune disease MDTs also helps support safe, efficient delivery of new novel therapies and can help establish governance and monitoring of increasingly complex treatment regimens.

Improving national coordination and investment in rare kidney disease research offers an opportunity to strengthen service provision and research capacity across the UK. The newly launched LifeArc-Kidney Research UK Centre for Rare Kidney Diseases, a £10.4 million initiative, for example, aims to accelerate research and innovation in rare kidney diseases, focusing initially on national collaboration and capacity building to support paediatric research [38].

A limitation of this survey is that it primarily captured the perspectives of nephrologists, whereas the management of glomerular disease in the UK is inherently multidisciplinary. Because clinical care and research involve a broad range of medical, nursing, and allied health specialties, these results may not fully reflect those broader viewpoints. Clinic frequency was also captured as a marker of service infrastructure across the UK rather than an assessment of adequacy relative to local demand as it was not adjusted for centre size, catchment population, or patient volume. Single weekly clinic may still adequately serve a smaller catchment area or, conversely, may indicate unmet capacity where local demand is high. Notably, only 1 of the 18 centres reporting more than one clinic per week identified as non-tertiary, consistent with clinic frequency scaling broadly with centre size rather than reflecting arbitrary variation in provision. Given the small paediatric sample, paediatric comments were also grouped descriptively into related categories of overlapping content, rather than analysed using formal inductive content analysis.

This survey primarily assessed equity of access at the service and centre level. However, some autoimmune glomerular diseases, such as lupus nephritis, disproportionately affect populations facing additional barriers to equitable care, including children accessing unlicensed or unfunded novel therapies, patients with genetic glomerular disease, those of lower socioeconomic status or with a first language other than English, and women of childbearing age. Future studies incorporating patient-level demographic and outcome data, potentially through linkage with national registries, would help characterise these dimensions of inequity more directly. It would also be useful to capture information on long-term follow-up practices, such as the extent to which centres have capacity to contribute longitudinal data to registries such as the National Registry of Rare Kidney Diseases.

Nationally, such targeted surveys could be used to understand variation in early access to specialist care with focused questions on patient triage into GN services and the availability of timely histology. These data can be used to inform the renal networks and support them in working with their integrated care boards to ensure equity of access to excellent care across their whole region.

## Conclusions

The rapidly evolving GN treatment landscape has exposed the need for subspecialist rare renal autoimmune services. This national survey highlights substantial variation in renal autoimmune disease services across the UK, with marked disparities in specialist staffing, clinic infrastructure, and access to novel therapies. Clinician engagement in research is strong; however, significant limitations are described with time and workforce capacity being critical. Addressing these imbalances could support closer alignment of renal services with the 2025 UK government mandate for rare autoimmune rheumatic diseases. Strategic investment, workforce planning, implementation of network-based service models, strengthened regional MDTs, and protected time for research are each essential to ensure that the full potential of emerging therapies for glomerular diseases is both realised and made equitably accessible to all patients with these conditions across the UK.

## Supplementary Information

Below is the link to the electronic supplementary material.

Supplementary Material 1

## Data Availability

The survey responses are available from the corresponding author on reasonable request.

## Abbreviations

AAV: ANCA-associated vasculitis
CKD: Chronic kidney disease
CSG: Clinical study group
ESKD: End stage kidney disease
GIRFT: Getting It Right First Time
GN: Glomerulonephritis
KRC GN CSG: Kidney Research Consortium Glomerulonephritis Clinical Study Group
IgAN: IgA Nephropathy
KDIGO: Kidney Disease: Improving Global Outcomes
KRC: Kidney Research Consortium
LN: Lupus Nephritis
NICE: National Institute for Health and Care Excellence
MDT: Multidisciplinary team
PAs: Programmed activities
RAIRDA: Rare Autoimmune Rheumatic Disease Alliance
SPIN: General paediatricians with a special interest in nephrology
UKIVAS: United Kingdom and Ireland Vasculitis Rare Disease Group
VOICES: Vasculitis Outcomes in Relation to Care Experiences
