# Supplementary material for: A national survey of autoimmune glomerular disease services in the UK
Source: BMC Nephrol. 2026 Aug 5;27:643. doi: 10.1186/s12882-026-05257-6 (PMC13729342; doi:10.1186/s12882-026-05257-6)
Supplement: Supplementary file 1 [file 12882_2026_5257_MOESM1_ESM.docx]

**Supplementary information/Appendix**

1. **Glomerular Disease, Renal Vasculitis and Lupus Services in the UK Survey**

This is a short survey for the Glomerulonephritis Clinical Study Group. We are looking at the variation in glomerular disease, renal vasculitis and lupus services in the UK. The questions are specifically about GN/renal vasculitis and lupus services. The aim is to gather and analyse current state of affairs in order to understand unmet needs nationally.

1. Your name
2. Your job title
3. Your renal unit name
4. Is your unit tertiary referral?
5. What is your regional renal network?

5a. Please specific your local renal network name:

5b. What trust does your renal unit belong to?

6. Do you have specialist clinics for the following: primary GN. Vasculitis, lupus

6.a. If not listed above, do you have any other specialist clinics or combination/s of the above?

7. How many clinics a week?

7.a. If less than 1 a week, how often?

8. Do you have consultants that sub specialise in these diseases? If so, how many?

8.a. Please describe your consultant's working model for managing these patients

9. Do you have Specialist nurse(s)? If so, how many?

10. Do you have research nurses? If so, how many?

11. Do you have dedicated advice phone line or messaging service?

12. Do you have an infusion service?

13. Do you have a plasma exchange service?

14. Do you have an in house MDT?

14.a. How often is your MDT?

14.b. Which other specialities attend your MDT meetings?

15. Do you have regional MDT?

15.a. If so, how many hospitals are involved?

16. Do you have Blueteq access for: Belimumab,Avacopan,Voclosporin,Rituximab?

16.b. Any other medication that you have Blueteq access for?

16.c. If you do not use Blueteq, what are your alternative arrangements for accessing new high cost therapies?

17. What would help improve your service?

18. Any other comments?

**Research related questions**

19. Does your renal unit run commercial clinical trials in these diseases?

20. Do you run academic clinical studies in these diseases?

21. Would you like to do more research

22. If so what are the challenges?

23. If you do not do clinical trials do you refer your patients to another hospital?

24. What would be your preferred model?

25. Any other research related comments?
